# The Influence of High-Concentrate Diet Supplemented with Tannin on Growth Performance, Rumen Fermentation, and Antioxidant Ability of Fattening Lambs

**DOI:** 10.3390/ani14172471

**Published:** 2024-08-25

**Authors:** Lu Lin, Yuezhang Lu, Weiqian Wang, Wenjun Luo, Tao Li, Guang Cao, Chunmei Du, Chen Wei, Fuquan Yin, Shangquan Gan, Jian Ma

**Affiliations:** 1 College of Coastal Agricultural Sciences, Guangdong Ocean University, Zhanjiang 524088, China; 11331319ww@stu.gdou.edu.cn (L.L.); 11331216jj@stu.gdou.edu.cn (Y.L.);; 2 Tie Qi Li Shi Feed Co., Ltd., Chengdu 610021, China

**Keywords:** lamb, tannin, nutrient digestibility, immunity, antioxidant ability

## Abstract

**Simple Summary:**

Lamb fattening is the main production model of mutton sheep farming because it can produce high-quality meat. In order to meet the requirements of nutrients, the fattening lambs are often provided a high-concentrate diet for maintaining productivity. However, during the lamb period, the functions of the immune and digestive organs are relatively low. Long-term feeding of a high-concentrate diet further aggravates the damage to the gastrointestinal tracts and the oxidative stress of lambs. Thus, alleviating oxidative stress and promoting healthy growth of fattening lambs are of great significance in the intensive feeding of the mutton sheep industry. Our experiment studied the effects of tannin supplementation on the growth performance, rumen fermentation, apparent digestibility, and serum antioxidant and immune indexes in fattening lambs fed a high-concentrate diet. The results of this study indicate that tannin can be used as an effective additive to improve immunity and antioxidant ability in fattening lambs.

**Abstract:**

This experiment aimed to study the effects of tannin supplementation on growth performance, rumen fermentation characteristics, apparent digestibility and serum biochemistry, and antioxidant and immune indexes in fattening lambs. A total of 36 male Hu sheep lambs (body weight = 15.83 ± 0.48 kg and days of age = 55 ± 2 d) were fed a high-concentrate diet and randomly divided into one of three groups of 12 animals each: control with no tannin (CON) and tannin treatments (TA1, 3 g/d per lamb; TA2, 6 g/d per lamb). The feeding experiment lasted for 60 d. The results showed that the average daily gain and ruminal propionate content of lambs in the TA1 group were higher (*p* < 0.05) than those in the CON group. Lambs fed tannin had significantly increased (*p* < 0.05) microbial protein and decreased (*p* < 0.05) ammonia nitrogen concentrations in the rumen. In addition, the crude protein and neutral detergent fiber digestibility of the TA2 group were significantly decreased (*p* < 0.05) as compared with the TA1 and CON groups, respectively. The serum concentrations of triglyceride, immunoglobulin A, and catalase and the total antioxidant capacity were higher (*p* < 0.05) in the TA1 group that those in the CON group, whereas an opposite trend of urea nitrogen, interleukin-1β, and malondialdehyde was found between the two groups. Also, tannin supplementation increased (*p* < 0.05) *Lactobacillus* and decreased (*p* < 0.05) *Salmonella* counts in the feces of lambs. Taken together, tannin supplementation can improve the growth performance, immunity, and antioxidant ability of fattening lambs fed a high-concentrate diet.

## 1. Introduction

With the improvement in living standards and pursuit in healthy diet, the consumption structure of meat has gradually changed. In recent years, the proportion of mutton consumption has increased significantly. As an important food source for humans, mutton is rich in a variety of nutrients, including high-quality protein, fatty acid, vitamins, and minerals, and plays an important role in promoting health, enhancing immunity, and providing energy to the body [1]. This increase in the demand for mutton consumption promotes the development of the meat sheep breeding industry. The feeding regimen is a critical factor to determine the quality and consumer acceptance of mutton [2]. Although pasture feeding is cost-effective, as well as environmentally friendly, a feed-based system can decrease the fattening time and regulate the fatty content of meat to satisfy the demand of people for high-quality mutton [2]. Moreover, grassland resources are limited in some areas of the world, and the demand for grassland ecological conservation and restoration is urgent. The traditional grazing management of the sheep industry is being gradually replaced by indoor feeding [2]. Under stall-feeding conditions, because of the limited moving space and feed type changes, some adverse impacts will be generated for sheep (e.g., reduced resistance to oxidative stress and a reduction in immunity), which restrain the development of the sheep industry [3]. Lamb fattening is a main production model of mutton sheep farming because it can shorten the time to market and produce high-quality meat. However, during the lamb period, the functions of the immune and digestive organs are relatively low. In the process of fattening lambs, a high proportion of the concentrate in the diet is commonly provided to meet the requirements of various nutrients for maintaining productivity, which further causes oxidative stress to the lambs [4]. In ruminants, oxidative stress is an important factor leading to immune impairment and inflammatory response [5]. Thus, alleviating oxidative stress and promoting the healthy growth of fattening lambs have significant meanings in intensive feeding in the mutton sheep industry.

In animal production, bioactive substances derived from natural plants, such as polyphenols, have been widely used to improve nutrient digestion, immunity, and gastrointestinal function, which have a beneficial influence on the growth performance and productivity of animals [6,7]. Tannin, a phenolic compound from plant secondary metabolism, is the second most abundant plant phenolic after lignin and can be used as a natural feed additive with its multiple antioxidant, anti-inflammatory, anticancer, and antimicrobial biological abilities [8]. With the banning of antibiotics and some drug feed additives in the ration, tannin has been gradually utilized in animal farming as an alternative to antibiotics. In lactating Holstein cows, the dietary supplementation of chestnut tannin at the level of 30 g/d can enhance the blood antioxidant enzyme activities and reduce nitrogen excretion in the urine, thus improving the milking performance [9]. In young ruminants, the addition of tannin in the diet increased the average daily gain (ADG), as well as the dry matter intake (DMI) of dairy calves [10]. Tannins can be divided into hydrolyzable and condensed tannins based on their chemical structures and properties. Compared with condensed tannin, the complex formed by hydrolyzable tannin and protein is easily hydrolyzed to low-molecular-weight polyphenols such as gallic acid and ellagic acid, which can be absorbed by animals [11]. Tannin is often used as a feed antioxidant to relieve some physiological stress to the animals because of high levels of plant polyphenols. Previous research in heat-stressed lambs reported that tannin supplementation enhanced the serum and liver superoxide dismutase (SOD) and glutathione peroxidase (GSH-Px) activities, thus improving the antioxidant capacity of lambs, which was helpful for alleviating heat stress [12]. Nevertheless, the positive effects of tannin on the nutrient digestibility, immunity, and antioxidant ability of fattening lambs under high-concentrate feeding conditions have not been fully explored.

The gastrointestinal tract plays an essential role in the nutrient digestion and immune function of ruminants. As a vital organ for volatile fatty acid (VFA) production and absorption, the rumen is strongly related to the growth performance and digestibility of ruminants [13]. During the fattening process, long-term feeding of a high-concentrate diet results in metabolic disorders of the gastrointestinal tracts, which can decrease the immunity, antioxidant capacity, and growth performance of animals [14]. Tannin can condense with various nutrients, such as protein, vitamins, and mineral elements, through hydrophobic or hydrogen bonds to form stable tannin–nutrient complexes, which can effectively protect nutrients from being decomposed and utilized by rumen microorganisms. Moreover, tannin can regulate the quantity of archaea, especially methane bacteria, which is conducive to reducing the production and emission of methane [15]. As mentioned earlier, long-term high-concentrate feeding can induce oxidative stress and inflammatory reactions and weaken the immunity of ruminants. Previous studies in calves, cows, and lambs have found that the dietary addition of tannin can improve gut health, antioxidant ability, and growth performance [9,10,16]. Although tannin can increase the antioxidant ability and productivity of animals, the information regarding tannin as an effective additive in fattening lambs fed a high-concentrate diet is limited. Thus, in the current experiment, we hypothesized that dietary supplementation of tannin may improve the antioxidant ability and immunity of fattening lambs. This study was performed to investigate the effects of tannin supplementation on the growth performance, rumen fermentation, apparent digestibility, and fecal microbial count, as well as the serum biochemistry and antioxidant and immune indexes, in fattening lambs fed a high-concentrate diet.

## 2. Materials and Methods

### 2.1. Experimental Design and Feeding Management

All experimental procedures involving animal care and management were authorized by the Institutional Animal Care and Use Committee of Guangdong Ocean University (Approval Code: SYXK-2023-086). The animal study was performed according to the Regulation on the Administration of Laboratory Animals (2017 Revision) promulgated by the State Council.

In the present research, a total of 36 male Hu lambs with similar body weight (BW, 15.83 ± 0.48 kg) and days of age (55 ± 2 d) were randomly selected. All the lambs were marked with ear tags and divided into one of three groups with 12 animals each. One group served as the control without tannin supplementation (CON), and the others were tannin groups that were supplemented with tannin (Wufeng Chicheng Biotechnology Co., Ltd., Yichang, China; hydrolyzable tannin ≥ 78%) at the levels of 3 g/d (TA1) and 6 g/d (TA2) per lamb. The addition levels of tannin were based on a previous study of heat-stressed lambs [12] and the recommendations of the manufacturer for ruminants.

All experimental animals of the three groups were housed in individual stalls (2 m × 1 m). The lambs were fed the same experimental diet twice daily at 08:30 and 18:30 and had access to clean water that was freely consumed. During the experimental period, the DMI was approximately 1.32 kg/d per lamb. At the morning feeding, tannin was fully blended with the basal diet to feed animals to ensure complete intake. The basal diet with a 30:70 roughage to concentrate ratio was formulated based on the NRC [17]. The concentrates mainly included corn, soybean meal, wheat bran, and cottonseed meal, and the roughages were alfalfa hay and corn straw. The feed compositions and nutrient levels of the experimental rations are described in Table 1. After a 10-day adaptive phase, the experimental period lasted for 60 days.

### 2.2. Determination of Growth Performance

The BWs of each lamb were measured with an electronic scale (Yuan Yueheng Electronic Technology Co., Ltd., Chengdu, China) on days 0 and 60 before the morning feeding during the experimental period and referred to as the initial and final BW, respectively, which were used to calculate the ADG. In addition, the difference between the feed offered and the orts was recorded to obtain the feed intake and subsequently convert it into the DMI. Through dividing the DMI by the ADG, the feed conversion ratio of each lamb was obtained.

### 2.3. Sample Collection of Blood, Ruminal Fluid, and Feces

Blood samples from each lamb were collected from the jugular vein by using 5 mL vacuum tubes (Shengcixing Trading Co., Ltd., Hengshui, China) before morning feeding on day 60 of the trial. Then, the serum was separated by centrifuging the blood samples at 3200 rpm for 12 min and placed into 1.5 mL centrifugal tubes (−20 °C) for further measurement. In addition, on day 60, using a stomach tube (Huazhi Kaiwu Technology Co., Ltd., Chengdu, China), the rumen fluid of each lamb was obtained at 4 h after the morning feeding. Next, the pH was measured with a pH meter (Wanbang Environmental Protection Technology Co., Ltd., Shenzhen, China). After pH determination, the fluid samples were filtered by nylon cloth and put in 5 mL centrifugal tubes (−20 °C) for subsequent analysis.

The feces of all animals were collected from days 55 to 60 of the experimental period by nylon sieve plates under the floor of the corresponding stalls. At the same time, the offered feed and the orts of each animal were collected. The collected feces were fully mixed for 6 consecutive days and weighed. Next, 10% of the total feces was collected for drying. In addition, a part of the feces of each lamb was placed into a 5 mL sterile tube (−80 °C) for the analysis of the microbial count. The fresh diet, orts, and feces of each lamb were dried (65 °C) in a drying oven (Zhengyuan Equipment Technology Co., Ltd., Shenzhen, China) to a constant weight. Then, the air-dried samples were ground to pass through a 1 mm sieve for nutrient digestibility determination.

### 2.4. Analysis of Rumen Fermentation Characteristics and Serum Indexes

First, the ruminal fluid samples were thawed. Then, 5 mL of ruminal fluid samples were blended with 1 mL of 25% metaphosphate. The mixture was centrifuged at 15,000 rpm and 4 °C for 10 min to collect the supernatant. Next, the supernatant was used to analyze the concentrations of VFA using a gas chromatograph (GC-2010) equipped with a capillary column (30 mm × 0.32 mm × 0.25 mm) [18]. The parameters for the gas chromatograph inlet sample were as follows: carrier gas N_2_, 40:1 of split ratio, and 0.6 µL of injection volume. The detector parameters were FID with a temperature of 230 °C, 40 mL/min flow rate of H_2_, 450 mL/min flow rate of air, and 45 mL/min flow rate of make-up gas. The column oven temperature program was increased from 120 °C to 180 °C at the rate of 10 °C/min. The 460 μL of acetate, 360 μL of propionate, 290 μL of butyrate, 10 μL of isobutyrate, 20 μL of valerate, and 20 μL of isovalerate were accurately measured and placed in 100 mL volumetric bottles, and then the distilled water was fixed to the scale to obtain a mixed standard solution. In addition, another group of 10 mL ruminal fluid samples was centrifuged at 15,000 rpm and 4 °C for 10 min to collect the supernatant, which was utilized to determine the contents of ammonia nitrogen (NH_3_-N) and microbial protein (MCP) with the methods of phenol-sodium hypochlorite colorimetry [19] and Lowry’s assay [20], respectively.

After unfreezing on ice, the serum samples were centrifuged at 3000 rpm for 10 min at 4 °C to obtain supernatant. The concentrations of biochemical indicators in serum which include albumin (ALB), alkaline phosphatase (ALP), alanine transaminase (ALT), aspartate transaminase (AST), globulin (GLB), glucose (GLU), non-esterified fatty acid (NEFA), triglyceride (TG), total protein (TP), and urea nitrogen (UN) were determined with a biochemistry analyzer (BS-360S, Ali Road medical equipment Co., Ltd., Wuhan, China). In addition to biochemical indicators, the contents of antioxidant parameters, immunoglobulins, and cytokines including catalase (CAT), GSH-Px, malondialdehyde (MDA), SOD, total antioxidant capacity (T-AOC), immunoglobulin A (IgA), IgG, IgM, interleukin-1β (IL-1β), IL-6, IL-10, and tumor necrosis factor-α (TNF-α) in serum samples were measured with commercial kits (Nanjing Jiancheng Bioengineering Institute, Nanjing, China) following the procedures of the instructions.

### 2.5. Analysis of Apparent Digestibility and Microbial Count

The fresh feed, orts, and feces were used to analyze the contents of crude protein (CP, method 984.13), dry matter (DM, method 934.01), ether extract (EE, method 954.02), and organic acid (OM, method 942.05) according to the methods of AOAC [21]. In addition, the neutral detergent fiber (NDF) and acid detergent fiber (ADF) contents in the samples mentioned earlier were determined by an Ankom fiber analyzer. The apparent digestibility (AD) was calculated using the following equation: AD (%) = [(nutrient intake − nutrient content in feces)/nutrient intake] × 100.

In the current experiment, the number of *Lactobacillus*, *Bifidobacterium*, *Escherichia coli*, and *Salmonella* in the feces of all lambs was evaluated by the plate count method following the procedures of previous research [22,23]. In brief, 10 g of feces from lambs was dissolved in 90 mL sterile water with mixed uniformity. Next, the mixture was serially diluted to enumerate the microbial counts. The small protrusions that formed on the MRS plate with slightly white wet and neat edges were *Lactobacillus*, and the double concentric ring colonies that formed a central bulge on the BBL plate were regarded as *Bifidobacterium*. Small colonies with purplish black and metallic luster forming on the eosin methylene blue plate were deemed to be *Escherichia coli*, and the colonies with black centers forming on the SS medium were *Salmonella*.

### 2.6. Statistical Analysis

All the data were analyzed by one-way ANOVA procedures based on each lamb as the experimental unit in the SPSS software (version 22.0). A Duncan test was used to assess the differences among the three groups. The results were presented as means and standard error of the means. The significant difference was defined as *p* < 0.05, and 0.05 < *p* < 0.10 was considered as a tendency.

## 3. Results

### 3.1. Growth Performance

The differences in the growth performances in fattening lambs after tannin supplementation are shown in Table 2. No significant difference (*p* > 0.05) of the initial BW and DMI was observed among the three groups. However, the ADG of the TA1 group was higher (*p* < 0.05) than that of the CON group. Compared with the CON and TA2 groups, the ratio of DMI to ADG in the TA1 group was significantly reduced (*p* < 0.05). In addition, the final BW of the TA1 group tended to be higher (0.05 < *p* < 0.10) than that in the CON group.

### 3.2. Ruminal Fermentation

**Figure 1:**
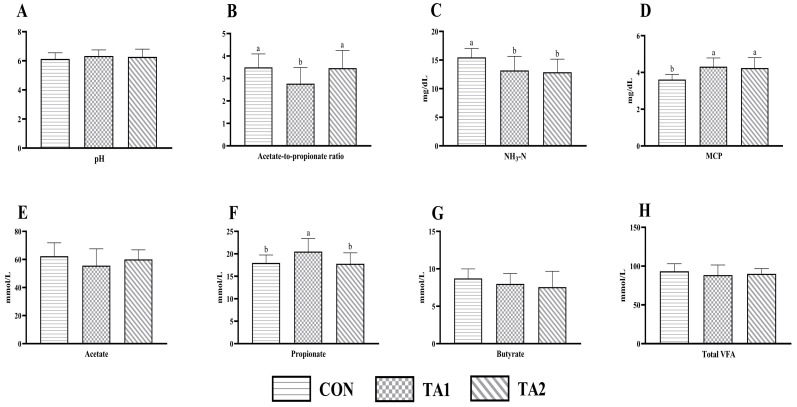
Effect of tannin supplementation on ruminal fermentation characteristics of fattening lambs. (**A**) pH, (**B**) acetate-to-propionate ratio, (**C**) NH_3_-N, (**D**) MCP, (**E**) acetate, (**F**) propionate, (**G**) butyrate, and (**H**) total VFA. NH_3_-N, ammonia nitrogen; MCP, microbial protein; VFA, volatile fatty acid. CON, TA1, and TA2 were fattening lambs fed the basal diet and supplemented with tannin at the dosages of 0, 3, and 6 g/d per animal, respectively. Columns with different small letters differ significantly (*p* < 0.05).

The ruminal pH was similar (*p* > 0.05) in the three groups with mean values of 6.13, 6.32, and 6.27, respectively (Figure 1A). Similarly, the concentrations of total VFA (Figure 1H), acetate (Figure 1E), and butyrate (Figure 1G) were not significantly different (*p* > 0.05) among the three treatment groups. Tannin supplementation significantly reduced (*p* < 0.05) the ruminal NH_3_-N content (Figure 1C) of lambs, whereas an opposite trend of MCP (Figure 1D) was found between CON and tannin treatments. Compared with the CON and TA2 groups, the propionate concentration (Figure 1F) in the TA1 group was increased (*p* < 0.05) by 14.20% and 15.35%, respectively. Conversely, the acetate-to-propionate ratio (Figure 1B) of the TA1 group was lower (*p* < 0.05) than that of the CON and TA2 groups.

### 3.3. Nutrient Digestibility

**Figure 2:**
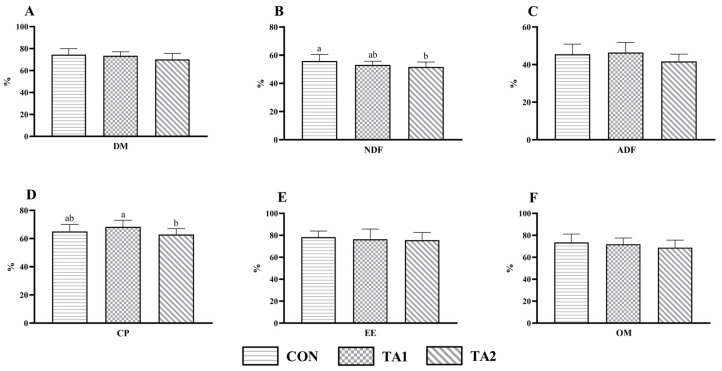
Effect of tannin supplementation on the nutrient digestibility of fattening lambs. (**A**) DM, (**B**) NDF, (**C**) ADF, (**D**) CP, (**E**) EE, and (**F**) OM. DM, dry matter; NDF, neutral detergent fiber; ADF, acid detergent fiber; CP, crude protein; EE, ether extract; OM, organic matter. CON, TA1, and TA2 were fattening lambs fed the basal diet and supplemented with tannin at the dosages of 0, 3, and 6 g/d per animal, respectively. Columns with different small letters differ significantly (*p* < 0.05).

There was no significant difference (*p* > 0.05) in the apparent digestibility of EE (Figure 2E) and OM (Figure 2F) among the three groups. However, the NDF digestibility (Figure 2B) of the TA2 group was significantly decreased (*p* < 0.05) when compared with that of the CON group. Also, the CP digestibility (Figure 2D) of the TA2 group was lower (*p* < 0.05) than that of the TA1 group. Additionally, the DM digestibility (Figure 2A) of the TA2 group was slightly lower (0.05 < *p* < 0.10) than that of the CON group. Compared with the TA2 group, the ADF digestibility (Figure 2C) of the TA1 group was increased (0.05 < *p* < 0.10) by 11.39%.

### 3.4. Biochemical Index of Serum

As shown in Table 3, the concentrations of ALP, ALT, AST, TP, GLB, ALB, NEFA and GLU in serum were not different (*p* > 0.05) among all groups. However, the serum TG content of lambs was significantly increased (*p* < 0.05) after tannin intervention, while an opposite trend of UN content was observed between the CON and tannin treatments.

### 3.5. Immunoglobulin and Cytokine of Serum

There were no obvious differences (*p* > 0.05) in the IgM and IL-6 serum concentrations among the three groups (Table 4). The serum IgA content of the TA1 group was higher (*p* < 0.05) than that of the CON group, while the opposite was found for IL-1β between the CON and TA1 groups. Compared with the CON group, a slight elevation (0.05 < *p* < 0.10) of serum IgG content was found in the TA1 group, while an opposite tendency of TNF-α was observed between the CON and tannin groups. Moreover, the IL-10 concentration in the TA2 group was slightly increased (0.05 < *p* < 0.10) when compared with that in the CON group.

### 3.6. Antioxidant Parameter of Serum

Obviously, the CAT concentration in the serum of the TA1 group was higher (*p* < 0.05) than that of the CON group (Table 5). Compared with the CON group, the GSH-Px concentration of the TA2 group was increased (0.05 < *p* < 0.10) by 8.34%. The SOD activity was similar (*p* > 0.05) among all groups. Lambs fed tannin had significantly increased (*p* < 0.05) serum T-AOC concentrations. Furthermore, tannin supplementation significantly reduced (*p* < 0.05) the MDA activity in the serum of lambs.

### 3.7. Microbial Count of Feces

**Figure 3:**
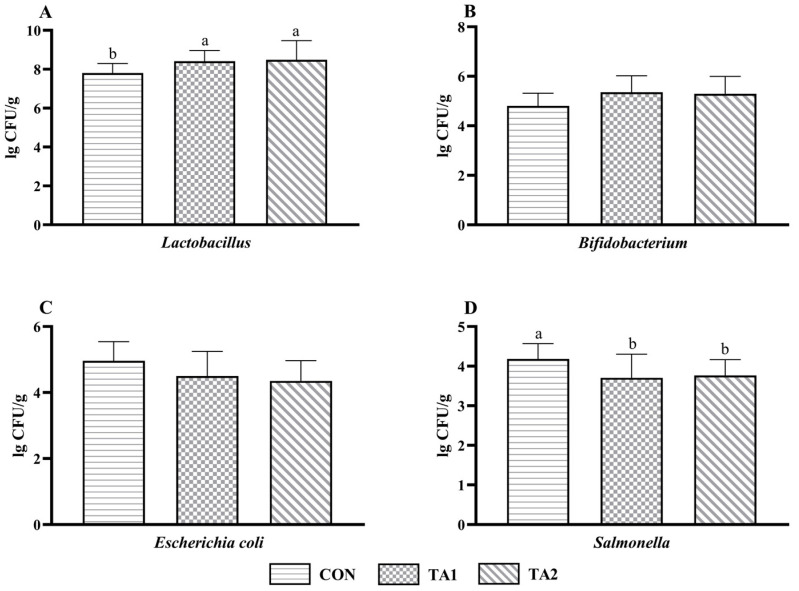
Effect of tannin supplementation on microbial count in the feces of fattening lambs. (**A**) *Lactobacillus*, (**B**) *Bifidobacterium*, (**C**) *Escherichia coli*, and (**D**) *Salmonella*. CON, TA1, and TA2 were fattening lambs fed the basal diet and supplemented with tannin at the dosages of 0, 3, and 6 g/d per animal, respectively. Columns with different small letters differ significantly (*p* < 0.05).

Evidently, tannin supplementation significantly increased (*p* < 0.05) the *Lactobacillus* count (Figure 3A) in the feces of the lambs. The TA1 lambs tended to have a higher (0.05 < *p* < 0.10) *Bifidobacterium* count (Figure 3B) as compared with the CON lambs. In addition, the number of *Escherichia coli* (Figure 3C) in the TA2 group was slightly lower (0.05 < *p* < 0.10) than that in the CON group. Lambs fed tannin displayed a markedly decreased (*p* < 0.05) *Salmonella* count (Figure 1D) in their feces.

## 4. Discussion

In recent years, an important feature of the lamb market has been the elevated requirement for a high quality of mutton, which has promoted the rapid development of fattening lambs. The fattening lamb is characterized by a high growth rate and feed conversion, as well as improved meat quality, and healthy feeding of fattening lambs is of great significance for the improvement of meat quality and productivity [24]. In order to meet the requirement of nutrients, fattening lambs are often provided more concentrates. Nevertheless, the high-concentrate diet causes digestive dysfunction, which can result in the occurrence of oxidative stress and inflammatory responses, thereby decreasing the feed utilization and growth rate of ruminants [25]. Tannin is rich in plant polyphenol and has various physiological functions, which include antioxidative, antimicrobial, and anti-inflammation effects [8]. In lambs, research found that the inclusion of natural tannins from *Mimosa tenuiflora* hay increased the ADG, as well as the DMI [26]. In our study, the ADG of the animals was significantly increased after dietary supplementation of tannin at the level of 3 g/d, but the DMI did not show a significant difference. The different results in the feed intake may be because the experimental diet was distinct. In addition, our finding showed that the feed conversion ratio of lambs was improved in the TA1 group. A recent study in calves reported that the feed conversion ratio was enhanced by tannin supplementation [27], which was in accordance with our result. Plant polyphenols, the main ingredients of tannin, can oxidize proteins on the surface of bacteria and destroy the structure and function of protein molecules, thereby leading to the death of bacteria [28]. The beneficial influence of polyphenols mentioned earlier is conducive to inhibiting the growth of harmful bacteria [28], which can decrease the incidence rate and attenuate the negative impact on the growth performance of lambs caused by long-term high-concentrate feeding.

In the rumen, NH_3_-N provides a nitrogen source for the growth of ruminal microorganisms, which can reflect the dynamic balance between dietary protein degradation and microbial protein synthesis [24]. A previous study has found that tannin supplementation reduced the NH_3_-N concentration in the rumen of sheep [29]. Consistent with the previous study, our result showed that dietary supplementation of tannin decreased the ruminal NH_3_-N content of lambs, indicating that tannin could improve the utilization of NH_3_-N to some extent. The reason may be that tannin increases the uptake and utilization of NH_3_-N by rumen microorganisms, resulting in a decrease in rumen NH_3_-N concentration. In addition, the ruminal microorganisms can synthesize MCP with ammonia and other nutrients as materials [24]. Interestingly, tannin supplementation significantly increased the MCP concentration in the rumen of lambs, which matched our NH_3_-N result. Tannin has the ability to bind proteins through hydrogen to form a tannin–protein complex, which is stable in the rumen and resistant to ruminal microbial degradation [30]. Thus, tannins can reduce the amount of digested protein in the rumen and increase the flow of protein to the small intestine, as well as improving rumen MCP concentration.

In ruminants, the VFA, which provides most of the energy requirements, is an important energy source to maintain the metabolism and growth performance [13]. Our study found that the propionate concentration in the rumen of lambs was higher in the TA1 group than that in the CON and TA2 groups, suggesting that the appropriate supplementation of tannin can increase the ruminal propionate concentration. A recent study in Tan lambs has verified that dietary supplementation of tannin increased the ruminal propionate content [31], which was in line with our research. As a critical energy source, the GLU required by the body for metabolism mainly comes from gluconeogenesis in the liver, and propionate is a primary precursor for liver gluconeogenesis [32]. As a result, the increase in propionate in the rumen is beneficial for promoting healthy growth of fattening lambs. We also found that the addition of tannin at the level of 3 g/d significantly reduced the ruminal ratio of acetate to propionate, which suggested that tannin can regulate the ruminal fermentation type, and a propionate-type fermentation was occurring in lambs. Inconsistent with our result, a study in dairy cows found that supplementation of tannin did not have a significant effect on the ruminal acetate-to-propionate ratio [9], which might be that the physiological stage of animals was different. In the rumen, the propionate-type fermentation can provide more energy for the body and reduce the production of methane, thus reducing the energy consumption caused by belching, which is conducive to the weight gain of lambs. A previous study has demonstrated that tannin displays the ability to inhibit the growth of methanogens [15]. When the reproduction of methanogens is inhibited, the fiber-degrading bacteria can use hydrogen competitively, thereby reducing methane emissions and increasing propionate production. However, the effects of tannin supplementation rumen microbial composition still need in-depth investigations. The results from current study indicated that the appropriate addition of tannin (3 g/d per lamb) could improve the rumen fermentation of fattening lambs.

Nutrient apparent digestibility is an important indicator related to the growth performance of animals. Previously, an experiment in lambs found that with the elevation of dietary supplementation level of tannin, the apparent digestibility of DM, CP, and OM was linearly reduced [33]. In our study, the CP and DM digestibility was reduced in the TA2 group, indicating that the high level of tannin supplementation had a negative influence on the nutrient digestibility, which was basically in accordance with previous research [33]. Nevertheless, we found that the CP digestibility of the TA1 group was significantly increased as compared with the TA2 group. The positive role of tannin in improving CP digestibility may be attributed to the potential advantage of improvement in the ruminal MCP content, which was easily absorbed by the small intestine to enhance CP digestibility. In addition, the tannin–protein complex can increase the rumen-protected protein content and reduce the degradation of dietary protein by rumen microbiota [11]. After the tannin–protein complex enters the abomasum and intestine, it gradually degrades under the action of various enzymes and finally increases the protein utilization of animals [11]. Also, tannin can attach to the surface of protein-decomposing bacteria and reduce the proportion of degradable protein in the rumen, thereby promoting the absorption and utilization of protein in the intestine [34]. In addition to CP, the NDF digestibility of lambs was reduced after high levels of tannin supplementation. A previous study in Holstein bulls found that high levels of tannin supplementation (the basal diet supplemented with tannin at 0.9% and 2.7%) significantly reduced DM and CP digestibility but had no obvious effects on NDF, ADF, and EE digestibility [34]. The reduction in NDF digestibility in our study may be because the extracellular enzyme secreted by hydrolyzable tannin binds to the cell wall of fibrolytic bacteria in the rumen of lambs and then destroys the complete structure of the bacterial cell membrane and inhibits the activity of bacterial secretion of fibrolytic enzyme [35], which finally decreases the fiber digestibility. However, the potential mechanism of action requires further exploration.

Serum biochemical indexes are important parameters reflecting changes in the health and function of various organs in animals farming [36]. In the current study, no significant difference of ALP, ALT, and AST contents in serum was found among all groups, suggesting that tannin supplementation did not have a negative impact on the hepatic function of the lambs. The changes in the serum GLU, TG, and NEFA contents reflect lipid and energy metabolism [36]. Our finding showed that tannin supplementation significantly increased the serum TG concentration of lambs. The probable reason for the improvement in the energy metabolism of fattening lambs is that tannin, as a plant polyphenol, may improve lipid metabolism by regulating the composition of the gut microbiota [37], which is helpful for relieving TG in the adipose tissue to decompose into NEFA, thus maintaining the serum TG content. But more experiments are needed to explore the potential molecular mechanism. In addition, the nitrogen metabolism can be partly evaluated by the changes of TP and UN contents in serum [13]. Our study found that the serum UN concentration of lambs was significantly decreased after dietary tannin supplementation, which indicated that the nitrogen conversion was improved by tannin supplementation. This may be because tannin can reduce the ability of the liver to absorb NH_3_-N by restraining protein hydrolysis in the rumen, as seen in the reduction in the ruminal NH_3_-N results after tannin treatment. Subsequently, the liver converts nitrogen into urea and enters the blood circulation [38]. Previously, an experiment demonstrated that the serum UN content of finishing Tan lambs was gradually reduced with the elevation of tannin supplementation dosage [31], which is in accordance with our finding. Overmuch nitrogen excretion of ruminants not only slows down the feed efficiency, especially for fattening animals, but also results in environmental pollution. The improvement of nitrogen utilization by tannin might be conducive to enhancing the nitrogen absorption of fattening lambs. In future research, the data of nitrogen intake and excretion in the feces and urine should be collected to obtain the information about tannin supplementation on nitrogen conversion in lambs.

In this study, the IgA and IgG concentrations in the serum of the TA1 group were higher than those of the CON group, indicating that tannin supplementation at the level of 3 g/d enhanced the immunity of fattening lambs to a certain degree. In a meta-analysis study, tannin supplementation at the level of 100 mg/kg basal diet can increase the serum IgG and IgM contents of animals [39], which is basically consistent with our study. Plant polyphenols can enhance the immune system function and resistance of the body to disease by accelerating the activity of white blood cells and improving antibody production, as well as the cellular immune response [40], which might explain the positive effects on the immunity of lambs caused by tannin supplementation. Long-term feeding of a high-concentrate diet often damages the barrier function of gastrointestinal tracts. As a result, some harmful substances (e.g., lipopolysaccharides) enter into the bloodstream through the damaged gastrointestinal epithelium, which results in the release of inflammatory factors and causes inflammatory reaction [41]. In the present experiment, tannin supplementation at the level of 3 g/d reduced the serum IL-1β and TNF-α concentrations of lambs, which suggested that tannin had the ability to attenuate the inflammatory response and promote the healthy growth of lambs. A previous study found that tannin can effectively restrain the activation of NLRP3 inflammasome through inhibiting the NF-κB signaling pathway, thus down-regulating the secretion of pro-inflammatory cytokines [42]. The positive effects of tannin on the inflammatory response might be due to the inhibition of the NF-κB signaling pathway. However, more experiments, including in vivo and in vitro, should be performed in the future to verify the influence of the NF-κB pathway in relieving inflammation by tannin.

Oxidative stress is an important factor that leads to immune impairment and inflammatory response. Long-term feeding of a high-concentrate diet can damage the antioxidant function of the liver and gastrointestinal tracts, which induces oxidative damage in ruminants [43]. The results from our experiment showed that dietary supplementation with tannin enhanced the activity of GSH-Px, CAT, and T-AOC in the serum of the lambs, indicating that tannin supplementation increased the antioxidant ability of the lambs. A previous study in lambs demonstrated that tannin product supplementation at the level of 5 g/kg basal diet can increase the activity of antioxidant enzymes under heat stress conditions [12], which was consistent with our research. When the body is subjected to oxidative stress, tannin can induce the expression of a series of protective genes, such as SOD and GSH-Px, to express more antioxidant enzymes, by blocking Nrf2 ubiquitination, promoting the binding of Nrf2 translocation nuclei with the antioxidant response element, then down-regulating the production of reactive oxide species and enhancing the antioxidant ability of the body [44]. As an oxidative stress index, the MDA can damage the cells’ integrity and can be used to reflect the degree of injury for cells and tissues [31]. Our experiment found that tannin significantly reduced the serum MDA concentration of lambs. Oxidative stress has adverse effects on growth performance and meat quality, including the meat color, tenderness, and odor, of ruminants [12]. The improvement in the antioxidant capacity of lambs by tannin might have beneficial impacts on the meat quality of fattening lambs fed a high-concentrate diet.

There are a large number of microorganisms inhabiting the gastrointestinal tracts of animals, and the intestinal microorganisms play many roles in digestion and metabolism, as well as host immunity. When the high-concentrate diet is fed for a long time, the concentrate ferments rapidly in the rumen of ruminants, causing gastrointestinal metabolic diseases, which can lead to a reduction in production efficiency [45]. *Escherichia coli* and *Salmonella* are the main pathogens that induce diarrhea and threaten the health of lambs. In the current experiment, tannin supplementation reduced the *Escherichia coli* and *Salmonella* counts, which suggested that tannin can protect the guts of lambs against disease. Consistent with our result, a previous study in transportation stress in goats found that dietary supplementation of tannin resulted in bacteriostasis for *Escherichia coli* [46]. Tannin can change the potential on both sides of the cell membrane through depolarization or hyperpolarization and then destroy the structure of the cell membrane of the bacteria. Also, tannin can form chelates with iron ions and destroy the cell wall integrity of bacteria [47]. These effects can effectively inhibit the reproduction of pathogenic bacteria. *Lactobacillus* plays an important probiotic role in mammalian health by maintaining microecological balance, producing nutrients, and inhibiting the growth of pathogenic bacteria [48]. In dairy calves, the increased *Lactobacillus* count has been verified to have a positive correlation with an improved ADG and feed conversion ratio [22]. Our microbial results displayed that tannin supplementation significantly increased the number of *Lactobacillus* in the feces of lambs, which was helpful for improving the growth performance. In the future, studies of the function of the microbiome through metagenomics and host function through transcriptomics are needed to obtain more information on the role of the microbial community in the guts of lambs and their responses to tannin.

## 5. Conclusions

The results obtained from this experiment provide evidence that tannin supplementation at the level of 3 g/d improves the ADG, feed efficiency, and CP digestibility of fattening lambs. A high-concentrate diet supplemented with tannin improves the ruminal fermentation of lambs, which is reflected in a decreased NH_3_-N content and increased MCP and propionate concentrations. Additionally, tannin supplementation contributes in a number of ways to the immunity and antioxidant capacity of fattening lambs, including decreases in the IL-1β, TNF-α, and MDA contents in the serum and *Escherichia coli* and *Salmonella* counts in the feces and increases in the IgA, GSH-Px, CAT, and T-AOC concentrations in the serum and the *Lactobacillus* count in the feces. Based on the results mentioned above, tannin is an effective feed additive to improve the immunity and antioxidant ability in fattening lambs fed a high-concentrate diet. However, the current study is a feeding experiment, and thus, some mechanisms of action cannot be fully investigated. In the future, a slaughter experiment should be performed to focus on the effects of tannin supplementation on meat quality and gastrointestinal function and microbiota of fattening lambs under high-concentrate feeding.

## Figures and Tables

**Table 1:** Compositions and nutrient levels of experimental diet (DM basis).

Ingredients, %	
Alfalfa hay	22.36
Corn straw	7.64
Corn	40.87
Wheat bran	10.64
Soybean meal	8.37
Cottonseed meal	7.45
NaCl	0.50
Limestone	0.31
NaHCO_3_	0.96
CaHPO_4_	0.40
Premix ^1^	0.50
**Nutrients levels**	
DM (%)	91.12
NDF (%)	29.32
ADF (%)	15.60
CP (%)	16.20
EE (%)	3.26
Starch (%)	28.16
Ash (%)	5.30
Ca (%)	0.84
P (%)	0.49
ME ^2^ (MJ/kg)	9.94

DM, dry matter; NDF, neutral detergent fiber; ADF, acid detergent fiber; CP, crude protein; EE, ether extract; ME, metabolizable energy. ^1^ The premix provided the following per kilogram of diet: Fe, 100 mg; Zn, 60 mg; Mn, 40 mg; Cu, 10 mg; I, 0.60 mg; Se, 0.40 mg; Co, 0.20 mg; VA, 5000 IU; VD, 1200 IU; VE, 40 IU. ^2^ ME was a calculated value; the other nutritional levels were measured values.

**Table 2:** Effect of tannin supplementation on growth performance of fattening lambs.

Items	Groups			SEM	*p*-Value
	CON	TA1	TA2		
Initial BW (kg)	17.40	17.36	17.53	0.117	0.841
Final BW (kg)	34.46	35.96	35.11	0.275	0.078
ADG (g/d)	284.31 ^b^	310.01 ^a^	293.06 ^ab^	4.319	0.042
DMI (kg/d)	1.33	1.30	1.34	0.021	0.753
Feed efficiency	4.69 ^a^	4.20 ^b^	4.59 ^a^	0.079	0.021

BW, body weight; ADG, average daily gain; DMI, dry matter intake; SEM, standard error of the mean. CON, TA1, and TA2 were fattening lambs fed the basal diet and supplemented with tannin at the dosages of 0, 3, and 6 g/d per animal, respectively. Feed efficiency = DMI/ADG. Means with different superscript letters in the same row differ significantly (*p* < 0.05).

**Table 3:** Effect of tannin supplementation on biochemical indexes in the serum of fattening lambs.

Items	Groups			SEM	*p*-Value
	CON	TA1	TA2		
ALP (U/L)	259.35	258.89	248.20	7.194	0.784
ALT (U/L)	16.97	17.36	17.72	0.347	0.689
AST (U/L)	86.66	88.65	86.89	1.559	0.857
TP (g/L)	56.39	59.56	56.74	1.056	0.418
GLB (g/L)	27.97	25.88	26.36	0.702	0.456
ALB (g/L)	28.42	33.67	30.38	1.330	0.272
TG (mmol/L)	0.374 ^b^	0.428 ^a^	0.419 ^a^	0.007	0.004
NEFA (mmol/L)	0.169	0.155	0.158	0.007	0.696
GLU (mmol/L)	4.02	4.05	3.89	0.082	0.732
UN (mmol/L)	3.51 ^a^	3.11 ^b^	3.03 ^b^	0.064	0.003

ALP, alkaline phosphatase; ALT, alanine transaminase; AST, aspartate transaminase; TP, total protein; GLB, globulin; ALB, albumin; TG, triglyceride; NEFA, non-esterified fatty acid; GLU, glucose; UN, urea nitrogen; SEM, standard error of the mean. CON, TA1, and TA2 were fattening lambs fed the basal diet and supplemented with tannin at the dosages of 0, 3, and 6 g/d per animal, respectively. Means with different superscript letters in the same row differ significantly (*p* < 0.05).

**Table 4:** Effect of tannin supplementation on immunoglobulin and cytokine contents in the serum of fattening lambs.

Items	Groups			SEM	*p*-Value
	CON	TA1	TA2		
IgM (mg/mL)	4.50	4.52	4.61	0.159	0.961
IgA (mg/mL)	23.29 ^b^	27.42 ^a^	25.70 ^ab^	0.592	0.013
IgG (μg/mL)	51.17	55.53	54.22	0.796	0.068
IL-1β (ng/L)	89.65 ^a^	83.60 ^b^	84.93 ^ab^	1.027	0.036
IL-6 (ng/L)	140.75	140.69	145.26	2.803	0.758
IL-10 (ng/L)	70.19	75.99	77.74	1.382	0.061
TNF-α (ng/L)	505.77	486.09	489.89	3.740	0.071

IgM, immunoglobulin M; IgA, immunoglobulin A; IgG, immunoglobulin G; IL-1β, interleukin-1β; IL-6, interleukin-6; IL-10, interleukin-10; TNF-α, tumor necrosis factor-α; SEM, standard error of the mean. CON, TA1, and TA2 were fattening lambs fed the basal diet and supplemented with tannin at the dosages of 0, 3, and 6 g/d per animal, respectively. Means with different superscript letters in the same row differ significantly (*p* < 0.05).

**Table 5:** Effect of tannin supplementation on antioxidant parameters in the serum of fattening lambs.

Items	Groups			SEM	*p*-Value
	CON	TA1	TA2		
CAT (U/mL)	45.82 ^b^	50.29 ^a^	48.58 ^ab^	0.652	0.014
GSH-Px (U/mL)	138.98	147.08	150.57	2.167	0.078
SOD (U/mL)	70.63	69.27	67.83	1.070	0.579
T-AOC (U/mL)	15.33 ^b^	19.11 ^a^	19.38 ^a^	0.531	0.001
MDA (mmol/mL)	4.44 ^a^	3.65 ^b^	3.63 ^b^	0.141	0.023

CAT, catalase; GSH-Px, glutathione peroxidase; SOD, superoxide dismutase; T-AOC, total antioxidant capacity; MDA, malondialdehyde; SEM, standard error of the mean. CON, TA1, and TA2 were fattening lambs fed the basal diet and supplemented with tannin at the dosages of 0, 3, and 6 g/d per animal, respectively. Means with different superscript letters in the same row differ significantly (*p* < 0.05).

## Data Availability

The data are available from the corresponding author.
